# Compositional Features and Bioactive Properties of *Aloe vera* Leaf (Fillet, Mucilage, and Rind) and Flower

**DOI:** 10.3390/antiox8100444

**Published:** 2019-10-01

**Authors:** Mikel Añibarro-Ortega, José Pinela, Lillian Barros, Ana Ćirić, Soraia P. Silva, Elisabete Coelho, Andrei Mocan, Ricardo C. Calhelha, Marina Soković, Manuel A. Coimbra, Isabel C. F. R. Ferreira

**Affiliations:** 1 Centro de Investigação de Montanha (CIMO), Instituto Politécnico de Bragança, Campus de Santa Apolónia, 5300-253 Bragança, Portugal; 2 Department of Plant Physiology, Institute for Biological Research “Siniša Stanković”, University of Belgrade, Bulevar Despota Stefana 142, 11000 Belgrade, Serbia; 3 QOPNA & LAQV-REQUIMTE, Department of Chemistry, University of Aveiro, 3810-193 Aveiro, Portugal; 4 Department of Pharmaceutical Botany, “Iuliu Hațieganu” University of Medicine and Pharmacy, Gheorghe Marinescu Street 23, 400337 Cluj-Napoca, Romania; 5 Laboratory of Chromatography, Institute of Advanced Horticulture Research of Transylvania, University of Agricultural Sciences and Veterinary Medicine, 400372 Cluj-Napoca, Romania

**Keywords:** *Aloe barbadensis* Mill., nutritional composition, neutral sugars, phenolic compounds, organic acids, antioxidant capacity, antimicrobial activity, tyrosinase inhibitory activity, cytotoxicity

## Abstract

This work aimed to characterize compositional and bioactive features of *Aloe vera* leaf (fillet, mucilage, and rind) and flower. The edible fillet was analysed for its nutritional value, and all samples were studied for phenolic composition and antioxidant, anti-inflammatory, antimicrobial, tyrosinase inhibition, and cytotoxic activities. Dietary fibre (mainly mannan) and available carbohydrates (mainly free glucose and fructose) were abundant macronutrients in fillet, which also contained high amounts of malic acid (5.75 g/100 g dw) and α-tocopherol (4.8 mg/100 g dw). The leaf samples presented similar phenolic profiles, with predominance of chromones and anthrones, and the highest contents were found in mucilage (131 mg/g) and rind (105 mg/g) extracts, which also revealed interesting antioxidant properties. On the other hand, the flower extract was rich in apigenin glycoside derivatives (4.48 mg/g), effective against *Pseudomonas aeruginosa* (MIC = 0.025 mg/mL and MBC = 0.05 mg/mL) and capable of inhibiting the tyrosinase activity (IC_50_ = 4.85 mg/mL). The fillet, rind, and flower extracts also showed a powerful antifungal activity against *Aspergillus flavus*, *A. niger*, *Penicillium funiculosum*, and *Candida albicans*, higher than that of ketoconazole. Thus, the studied *Aloe vera* samples displayed high potential to be exploited by the food or cosmetic industries, among others.

## 1. Introduction

*Aloe vera* (also known as *Aloe barbadensis* Mill.) is a flowering succulent plant of the family Asphodelaceae currently naturalized in many tropical and sub-tropical countries. In traditional medicine, it has been widely used for centuries to treat skin disorders and other ailments, as well as for its purgative effect [1]. Today, this species is used worldwide as a valuable ingredient for functional foods (such as healthy drinks and other beverages), cosmetics (including creams, lotions, soaps, and shampoos), and drugs (such as tablets and capsules) [2].

The dagger-shaped leaves are the most used part of the plant, in which two major fractions can be identified, namely the outer, photosynthetically active green cortex, usually known as rind, and the inner parenchyma, known as pulp or fillet. Furthermore, the leaf secretes two different exudates—the reddish-yellow latex produced by the pericyclic cells under the cutinized epidermis and the transparent, slippery mucilage or gel produced by the thin-walled tubular cells in the inner parenchyma [3,4]. The gel is approximately 98% moisture, and the non-aqueous remainder largely consists of acemannan (a bioactive acetylated glucomannan) and other polysaccharides, sugars, minerals, organic acids, and vitamins [1,4,5,6]. Traditionally, it is used topically to treat wounds, minor burns, and skin irritations and internally to treat constipation, coughs, ulcers, and diabetes, among other ailments [1,7]. The latex, on the other hand, contains hydroxyanthracene derivatives, including anthraquinone *C*- and *O*-glycosides, and is a natural drug well-known for its cathartic effect and also used as a bittering agent in alcoholic beverages [8,9].

Today, *Aloe vera* is produced on a large scale in many countries around the world to supply the still growing and economically important industry [1]. For production of *Aloe vera* gel, the leaves can be processed either by grinding the inner fillet after removing the rind (treated as bio-waste) and rinsing away the latex, or the whole leaf. However, in the second case, a subsequent filtration/purification step is required to remove unwanted constituents, especially those from latex [10,11]. The gel has been marketed fresh or in powdered concentrate and included in different formulations for food, health, medicinal, and cosmetic purposes [9,10,12].

Over the last few decades, the *Aloe vera* leaf has been the subject of several scientific studies that aimed to characterize its chemical and biological properties [4,5,6,13,14]. Still, there are compositional and bioactive parameters that deserve further study, and the flower remains an underexploited plant part. Moreover, a lack of information on the exact part of the plant analysed or even the species involved is common in many works. There are confusing descriptions, mostly about the inner part of the leaf, due to the different terms that have been used interchangeably, such as fillet, pulp, mucilage, gel, and parenchyma, among others. Technically, these terms do not refer to the same part, since fillet or pulp refer to the fleshy inner part of the leaf including the cell walls, while gel or mucilage refer to the viscous clear liquid contained within the parenchyma cells [4].

This comprehensive study was performed to evaluate and compare compositional and bioactive features of different parts of *Aloe vera*, namely leaf (which was divided into fillet, mucilage and rind) and flower. More specifically, it was intended to determine the nutritional and chemical composition of the edible fillet and the profiles in phenolic compounds of the four sample extracts, as well as their antioxidant, anti-inflammatory, antimicrobial, tyrosinase inhibition, and cytotoxic capacities. This will provide accurate and up-to-date research information on *Aloe vera*.

## 2. Materials and Methods

### 2.1. Chemicals

All standard compounds used for chromatographic quantifications (47885-U, 2-deoxyglucose, α, β, γ, and δ tocopherols, oxalic, quinic, malic, ascorbic, citric, and fumaric acids, from Sigma-Aldrich (St. Louis, MO, USA); tocol, from Matreya (Pleasant Gap, PA, USA); chlorogenic acid, *p*-coumaric acid, apigenin-6-glucoside, apigenin-7-glucoside, and luteolin-6-*C*-glucoside, from Extrasynthèse (Genay, France); and aloin, from Alfa Aesar (Ward Hill, MA, USA)) and bioactivity assays (trolox, kojic acid, dexamethasone, ellipticine, streptomycin, and ketoconazole, from Sigma-Aldrich) had a purity level of at least 95%. All other reagents were of analytical grade and purchased from common sources.

### 2.2. Plant Material

Freshly cut *Aloe vera* samples (leaves and flowers) of certified organic production were supplied by “aCoruela do Alentejo,” a company located in the parish of São Brás e São Lourenço, municipality of Elvas, Portugal. Twelve three-year-old leaves with about 10 cm × 45 cm were weighed, and the green rind was separated from the inner fillet with a knife. Then, the transparent slippery exudate consisting mainly of gel was collected from the mucilage layer of the outer leaf pulp adjacent to the rind. The weight of the different leaf parts was determined to calculate the weight percentage (wt%). A batch of each plant part (fillet, mucilage, rind, and flower) was then lyophilized (FreeZone 4.5, Labconco, Kansas City, MO, USA), reduced to a fine powder (~20 mesh), and homogenized to obtain a representative sample that was kept at −20 °C until analysis.

### 2.3. Compositional Analysis of Fillet

#### 2.3.1. Proximate Composition

The edible fillet was analysed for moisture, protein, fat, ash, and crude fibre contents following the AOAC procedures [15]. Briefly, the crude protein content (N × 6.25) was estimated by the macro-Kjeldahl method, using an automatic distillation and titration unit (Pro-Nitro-A, JP Selecta, Barcelona); the crude fat content was determined by Soxhlet extraction with petroleum ether; the ash content was determined by incineration in a muffle furnace at 550 ± 15 °C; and the crude fibre content was estimated by the Weende method through an acid/alkaline hydrolysis of insoluble residues. The available carbohydrate content was determined by the anthrone method [16]. The dietary fibre content was estimated by difference. The results were given as g per 100 g of plant material.

The energy value was calculated as follows: 4 × (g protein + g available carbohydrates) + 9 × (g fat) + 2 × (g dietary fibre) [17] and given as kcal per 100 g of plant material.

#### 2.3.2. Fatty Acids, Tocopherols, and Organic Acids

Fatty acids were analysed in a DANI GC 1000 (DANI instruments, Contone, Switzerland) equipped with a split/splitless injector and a flame ionization detector (FID) at 260 °C. The fatty acids obtained by Soxhlet extraction were methylated with methanol:sulphuric acid:toluene 2:1:1 (v:v:v) during at least 12 h in a bath at 50 °C and 160 rpm. Then, deionised water was added to obtain phase separation, and the fatty acid methyl esters (FAME) were recovered with diethyl ether. The upper phase was dehydrated and filtered through 0.2 μm nylon filters for injection. Chromatographic separation was performed on a Zebron-Kame column (30 m × 0.25 mm i.d. × 0.20 µm film thickness, Phenomenex, Torrance, CA, USA). The oven temperature program was as follows: the initial temperature of the column was 100 °C, held for 2 min, then a 10 °C/min ramp to 140 °C, 3 °C/min ramp to 190 °C, 30 °C/min ramp to 260 °C, held for 2 min [18]. The carrier gas (hydrogen) flow rate was 1.1 mL/min, measured at 100 °C. Split injection (1:50) was carried out at 250 °C. The identification was made by chromatographic comparison of the retention times of the sample FAME peaks with those of commercial standards (standard 47885-U). The results were processed using the Clarity 4.0.1.7 Software (DataApex, Podohradska, Czech Republic) and given as relative percentage of each fatty acid.

Tocopherols were analysed in a high-performance liquid chromatography (HPLC) system (Knauer, Smartline system 1000, Berlin, Germany) coupled to a fluorescence detector (FP-2020, Jasco, Easton, MD, USA) programmed for excitation at 290 nm and emission at 330 nm, following an previously described analytical procedure [19]. The samples (500 mg) were spiked with a BHT solution (10 mg/mL) and tocol (internal standard, 50 μg/mL) and homogenized with methanol (4 mL) by shaking in vortex (1 min) and then with hexane (4 mL). After that, a saturated NaCl aqueous solution (2 mL) was added, the mixture was homogenized, centrifuged (5 min, 4000× *g*) and the clear upper layer was collected. The extraction was repeated twice with hexane. The obtained extracts were dried under a nitrogen stream, redissolved in 2 mL of *n*-hexane, dehydrated, and filtered through 0.22 μm disposable syringe filters for injection. Chromatographic separation was performed in normal phase on a Polyamide II column (5 μm particle size, 250 × 4.6 mm; YMC, Kyoto, Japan). Elution was performed with a mixture of *n*-hexane and ethyl acetate (70:30, v/v). The detected compounds were identified by chromatographic comparisons with authentic standards (α, β, γ, and δ isoforms) and quantified using the internal standard method. The results were processed using the Clarity 4.0.1.7 Software and given as µg per 100 g of plant material.

Organic acids were analysed in a ultra-fast liquid chromatography system (Shimadzu 20 A series UFLC, Shimadzu Corporation, Kyoto, Japan) coupled to a photodiode array detector (UFLC-PDA), as previously described [20]. The samples (1 g) were stirred with meta-phosphoric acid (25 mL) for 45 min and filtered, first through Whatman No. 4 paper and then through 0.2 µm nylon filters. Chromatographic separation was achieved in reverse phase on a C18 column (5 μm particle size, 250 × 4.6 mm; Phenomenex, Torrance, CA, USA). The elution was performed with sulphuric acid (3.6 mM). PDA detection was carried out at 215 and 245 nm (for ascorbic acid). The detected compounds were identified and quantified by chromatographic comparison of the peak area with calibration curves obtained from commercial standards (oxalic, quinic, malic, ascorbic, citric, and fumaric acids). The results were processed using the LabSolutions Multi LC-PDA software and expressed in mg per 100 g of plant material.

#### 2.3.3. Sugars and Glycosidic-Linkage Composition

The fillet sample was dialysed with a membrane cut-off of 12–14 kDa to recover the high molecular weight (HMW) compounds. Neutral sugars of the initial and dialysed samples were analysed by gas chromatography-flame ionization detection (GC-FID) after conversion to their alditol acetates [21,22]. The quantification was carried out using 2-deoxyglucose as internal standard. Monosaccharides were released from polysaccharides with pre-hydrolysis of the samples using 0.2 mL of 72% (w/w) H_2_SO_4_ for 1 h at room temperature followed by 2.5 h hydrolysis in 1 M H_2_SO_4_ at 100 °C. After hydrolysis, the reduction (NaBH_4_) and acetylation (acetic anhydride using methyl imidazole as catalyst) of the monosaccharides were performed. The alditol acetates were analysed using a DB-225 column (30 m, 0.25 mm i.d., 0.25 µm film thickness) and a GC-FID PerkinElmer-Clarus 400 [23,24]. Free sugars were also quantified on the initial sample (not dialysed) using the same method, by omitting the hydrolysis in the abovementioned steps. The oven temperature program was as follows: 220 °C, hold for 7 min, to 240 °C at a rate of 5 °C/min. The temperature of injector was 220 °C, and the detector was 240 °C. Hydrogen was used as the carrier gas.

Glycosidic-linkage composition of the dialysed sample (HMW) was determined by GC-qMS of the partially methylated alditol acetates as previously described [22]. The polysaccharides were methylated using CH_3_I, hydrolysed (TFA 2M) and the resultant monosaccharides were reduced (NaBD_4_) and acetylated. The partially methylated alditol acetates (PMAAs) obtained were analysed by gas chromatography mass spectrometry (GC-qMS) on a Shimadzu GCMS-QP2010 Ultra [25]. The GC was equipped with an SGE HT5 (Supelco, Bellefonte, PA, USA) fused silica capillary column (30 m length, 0.25 mm i.d., and 0.10 μm of film thickness).

### 2.4. Bioactive Analysis of Fillet, Mucilage, Rind, and Flower

#### 2.4.1. Preparation of Hydroethanolic Extracts

The powdered fillet, mucilage, rind, and flower samples (~1 g) underwent solid-liquid extraction twice with a 80:20 (v/v) ethanol:water mixture (30 mL) for 1 h at room temperature, and the supernatants were then filtered through Whatman no. 4 filter paper [26]. Ethanol was separated from the filtrates in a rotary evaporator (Büchi R-210, Flawil, Switzerland), and the aqueous phase was lyophilized to obtain dried extracts.

#### 2.4.2. Analysis of Phenolic Compounds

The dried extracts (10 mg) were dissolved in a 80:20 (v/v) ethanol:water mixture (2 mL) and filtered through 0.22-μm disposable syringe filters. The analysis was performed in a HPLC-DAD-ESI/MS^n^ system (Dionex Ultimate 3000 UPLC, Thermo Scientific, San Jose, CA, USA), as previously described [27]. Chromatographic separation was made in a Waters Spherisorb S3 ODS-2 C18 column (3 µm, 4.6 mm × 150 mm; Waters, Milford, MA, USA). Double online detection was carried out with a diode array detector (DAD, using 280 and 370 nm as preferred wavelengths) and a Linear Ion Trap (LTQ XL) mass spectrometer (MS, Thermo Finnigan, San Jose, CA, USA) equipped with an electrospray ionization (ESI) source. Phenolic compounds were identified by comparison of their retention times and UV-vis and mass spectra with those obtained from standard compounds, when available; otherwise, compounds were tentatively identified comparing the obtained information with available data reported in the literature. For quantitative analysis, a calibration curve for each available phenolic compound standard (aloin A (280 nm: *y* = 3859.4*x* + 21,770, *R²* = 0.9996; and 370 nm: *y* = 7184.4*x* + 17,013, *R*² = 0.9996); chlorogenic acid (*y* = 168,823*x* – 161,172, *R*² = 0.9999); *p*-coumaric acid (*y* = 301,950*x* + 6966.7, *R*² = 0.9999); apigenin-6-glucoside (*y* = 107,025*x* + 61531, *R*² = 0.9989); apigenin-7-glucoside (*y* = 10,683*x* – 45,794, *R*² = 0.9906); and luteolin-6-*C*-glucoside (*y* = 4087.1*x* + 72,589, *R*² = 0.9988)) was constructed based on the UV signal. Quantification of the phenolic compounds that are not commercially available as standards was performed by using the most similar available standard molecule. The results were expressed as mg per g of extract.

#### 2.4.3. Evaluation of Bioactive Properties

##### Antioxidant Activity

The extracts antioxidant capacity was evaluated by the in vitro assays of oxidative haemolysis inhibition (OxHLIA), thiobarbituric acid reactive substances formation inhibition (TBARS), and β-carotene bleaching inhibition (β-CBI), following methodologies previously described [28,29]. Extract concentrations ranging from 5 to 0.0159 mg/mL were used. Trolox was the positive control.

OxHLIA assay—An erythrocyte solution (2.8%, v/v; 200 µL) was mixed with 400 µL of either extract solution in PBS, PBS solution (control), or water (for complete haemolysis). After pre-incubation at 37 °C for 10 min with shaking, AAPH (200 μL, 160 mM in PBS, from Sigma-Aldrich) was added, and the optical density (690 nm) was measured every 10 min in a microplate reader (Bio-Tek Instruments, ELX800) until complete haemolysis [28]. The results were expressed as IC_50_ values (µg/mL) at a Δ*t* of 60 min, i.e., extract concentration required to keep 50% of the erythrocyte population intact for 60 min.

TBARS assay—A porcine brain cell solution (1:2, w/v; 0.1 mL) was incubated with the extract solutions (0.2 mL) plus FeSO_4_ (10 µM; 0.1 mL) and ascorbic acid (0.1 mM; 0.1 mL) at 37 °C for 1 h. Then, trichloroacetic (28% w/v, 0.5 mL) and thiobarbituric (TBA, 2%, w/v, 0.38 mL) acids were added, and the mixture was heated at 80 °C for 20 min. After centrifugation at 3000× *g* for 10 min, the malondialdehyde (MDA)-TBA complexes formed in the supernatant were monitored at 532 nm (Specord 200 spectrophotometer, Analytik Jena, Jena, Germany) [29]. The results were expressed as EC_50_ values (µg/mL), i.e., extract concentration providing 50% of antioxidant activity.

β-CBI assay—A β-carotene-linoleic acid emulsion (4.8 mL) was mixed with the extract solutions (0.2 mL) and the absorbance was measured at 470 nm as soon as mixed (A_βT0_) and after 2 h of incubation at 50 °C (A_βT0_). The β-CBI capacity was calculated as follows: (A_βT2_/A_βT0_) × 100 [29]. The results were expressed as EC_50_ values (µg/mL).

##### Antimicrobial Activity

The extracts were tested against the Gram-negative bacteria *Staphylococcus aureus* (ATCC 11632), *Staphylococcus epidermidis* (clinical isolate Ibis 2999), *Staphylococcus lugdunensis* (clinical isolate Ibis 2996), *Micrococcus flavus* (ATCC 10240), and *Listeria monocytogenes* (NCTC 7973), and the Gram-positive bacteria *Escherichia coli* (ATCC 25922), *Pseudomonas aeruginosa* (ATCC 27853), and *Salmonella* Typhimurium (ATCC 13311), as described by Soković et al. [30]. The fungi *Aspergillus flavus* (ATCC 9643), *Aspergillus niger* (ATCC 6275), *Penicillium funiculosum* (ATCC 36839), *Candida albicans* (clinical isolate Ibis 475/15), *Trichophyton mentagrophytes* (clinical isolate Ibis 2979/18), *Trichophyton tonsurans* (clinical isolate Ibis16/17), *Microsporum gypseum* (clinical isolate Ibis 3277/18), and *Microsporum canis* (clinical isolate Ibis 2990/18) were tested as described by Soković and van Griensven [31]. The microorganisms were obtained from the Mycological laboratory, Department of Plant Physiology, Institute for Biological Research “Sinisa Stanković,” University of Belgrade, Serbia. Streptomycin and ketoconazole were the positive controls used for the antibacterial and antifungal activities, respectively. The results were given as minimum inhibitory (MIC) and minimum bactericidal (MBC) or fungicidal (MFC) concentrations (mg/mL).

##### Anti-Inflammatory Activity

The extract capacity to inhibit the lipopolysaccharide (LPS)-induced nitric oxide (NO) production by a murine macrophage cell line (RAW 264.7) was determined as nitrite concentration in the culture medium [32]. Dexamethasone was used as a positive control, and negative controls were performed without LPS. NO production was determined using a Griess Reagent System kit containing sulfanilamide, *N*-1-naphthylethylenediamine dihydrochloride (NED), and nitrite solutions. The results were expressed as EC_50_ values (µg/mL), i.e., extract concentration providing 50% of NO production inhibition.

##### Tyrosinase Inhibitory Activity

The extracts capacity to inhibit the tyrosinase activity was measured according to the method of Chen et al. [33], using l-3,4-dihydroxyphenylalanine (l-DOPA) as substrate. For each extract, four wells of a 96-well plate were designated as A, B, C, and D, and each one contained a reaction mixture (200 µL) as follows: (A) 66 mM PBS (pH 6.8; 120 µL) and mushroom tyrosinase in PBS (46 U/mL; 40 µL); (B) PBS (160 µL); (C) PBS (80 µL), tyrosinase (40 µL), and aqueous extract solution with 5% DMSO (40 µL); and (D) PBS (120 µL) and aqueous extract solution with 5% DMSO (40 µL). After incubation of the reaction mixtures for 10 min at room temperature, 2.5 mM l-DOPA in PBS (40 µL) was added and the plate was incubated again for 20 min. The absorbance was measured at 475 nm (SPECTROstar Nano, BMG Labtech, Ortenberg, Germany). A kojic acid solution (0.10 mg/mL) was used as positive control. The inhibition percentage of the tyrosinase activity was calculated as follows:

(1) $$I\left(\%\right)=\frac{\left(A-B\right)-\left(C-D\right)}{\left(A-B\right)}\times 100$$

IC_50_ values were then calculated from the obtained inhibition percentage values.

##### Cytotoxic and Hepatotoxic Activities

The extracts cytotoxicity was evaluated by the sulforhodamine B (from Sigma-Aldrich) assay against five human tumour cell lines (acquired from Leibniz-Institut DSMZ): metastatic melanoma (MM127), breast adenocarcinoma (MCF-7), non-small cell lung carcinoma (NCI-H460), cervical carcinoma (HeLa), and hepatocellular carcinoma (HepG2), as previously described [34]. The same assay was also used to evaluate the extracts hepatotoxicity against a non-tumour cell line (porcine liver primary cells, PLP2) [35]. Ellipticine was used as positive control. The results were expressed in GI_50_ values (μg/mL), i.e., extract concentration providing 50% of cell growth inhibition.

### 2.5. Statistical Analysis

All analyses were performed in triplicate, and the results were presented as mean ± standard deviation. All statistical tests were performed at a 5% significance level using SPSS Statistics software (IBM SPSS Statistics for Windows, Version 22.0, IBM Corp., Armonk, NY, USA:). The fulfilment of the one-way analysis of variance (ANOVA) requirements, specifically the normal distribution of the residuals and the homogeneity of variance, was tested by means of the Shapiro Wilk’s and Levene’s tests, respectively. Depending on the homoscedasticity, the dependent variables were compared using Tukey’s honestly significant difference (HSD; when homoscedastic, *p* > 0.05) or Tamhane’s T2 multiple comparison (when heteroscedastic, *p* < 0.05) tests.

Differences between two samples were analysed by applying a two-tailed paired Student’s *t*-test; significant differences were considered when the *p*-value was lower than 0.05.

A Pearson’s correlation was run in SPSS to evaluate whether there is a relationship between the identified compounds and the measured bioactivities.

## 3. Results and Discussion

### 3.1. Nutritional Composition of Fillet

*Aloe vera* fillet has been used in the food industry to develop functional foods such as beverages, milk, yogurt, jam, jellies, ice cream, and food supplements, as well as in edible fruit coatings. It can also be used to improve the quality of meat products [36] and is often commercialized as concentrated dry powder. In this study, the fillet sample corresponded to 58 ± 4% of the total leaf weight, while 31 ± 2% consisted of green rind.

The nutritional composition of *Aloe vera* fillet is presented in Table 1. This inner part of the leaf consists of 98 ± 1 g/100 g of moisture, the same amount that was found in the mucilage. Similar moisture contents (98–99 g/100 g) were previously reported [5,9,37]. Lower values were found in the rind and flower samples (87 ± 1 and 84 ± 1, respectively; Table S1).

Dietary fibre was a predominant macronutrient in *Aloe vera* fillet, with 50.1 ± 0.3 g/100 g dw, followed by available carbohydrates, which corresponded to 37.4 ± 0.3 g/100 g dw (Table 1). A slightly higher dietary fibre content (57.64 g/100 g dw) was described by Femenia et al. [5] in fillet samples of *Aloe vera* cultivated in Ibiza, Spain. In turn, the fraction corresponding to crude fibre was isolated through acid and alkaline digestion of the sample and may consist of cellulose and small amounts of hemicellulose and lignin [38], which do not dissolve in the used solutions of sulphuric acid and potassium hydroxide. This small amount of insoluble fibre may correspond to the cell walls of the parenchyma cells that contain the gel. A higher crude fibre content (12.95 g/100 g dw) was previously reported in *Aloe vera* samples from Coquimbo, Chile [39].

The fresh fillet revealed reduced levels of protein and fat and slightly higher amounts of ash (minerals) (Table 1). This sample was, therefore, characterized by a low energy value (269 ± 3 kcal/100 g dw). These values are lower than that previously reported for fat (4.21 g/100 g dw) and ash (15.37–17.64 g/100 g dw) but slightly higher for the protein content (3.72–7.26 g/100 g dw) [5,39]. Potassium and calcium were previously found as major minerals in fillet samples [5] and may contribute to the wound healing capacity of this medicinal plant. Such compositional variations can be justified by the different geographical and edaphoclimatic conditions where the *Aloe* samples were grown.

As shown in Table 1, oxalic, quinic, and malic acids were detected in the fillet. Malic acid was the most abundant, with a concentration of 97 ± 1 mg/100 g of fresh fillet and 5.75 ± 0.07 g/100 g of dried powder. This acid is a natural component of aloe gel and an excellent freshness indicator. It was also detected along with the other two acids in the mucilage sample (Table S1). In fact, it was more abundant in the mucilage collected from the vascularized layer of the leaf, but the amount of total organic acids found in fillet and mucilage did not differ significantly. Bozzi et al. [9] also detected malic acid in fresh *Aloe vera* gel and others, like citric, lactic, and succinic acids, in commercial gel powders. However, citric acid (which can be found in the rind; Table S1) is added to the concentrated powders as a natural preservative by adjusting the gel pH prior to its concentration and drying in order to improve flavour and prevent oxidation. In turn, lactic and succinic acids should be absent from these concentrates, since they are indicators of bacterial fermentation and enzymatic degradation [9]. As presented in Table S1, ascorbic acid was not detected in the fillet and mucilage samples, but it was found in the green rind. Fumaric acid, in turn, was detected in rind and flower.

Tocopherols are important fat-soluble chain-breaking antioxidants. As shown in Table 1, the four isoforms were detected in the fillet, and α-tocopherol (4.8 ± 0.1 mg/100 g dw) was the most abundant, followed by γ- and β-tocopherols. Therefore, a 100 g portion of dried fillet provides about 69%, 44%, and 32% of the recommended dietary allowances of vitamin E for children from 4–8 and 9–13 years old and individuals with 14 or more years old, respectively (values calculated based on the α-tocopherol content) [40]. Bashipour and Ghoreishi [41] obtained a lower amount (1.53 mg/100 g dw) of α-tocopherol from *Aloe vera* samples grown in Isfahan, Iran, when applying optimized supercritical CO_2_ extraction conditions. Comparable α-tocopherol levels (4.70 mg/100 g dw) were reported by López-Cervantes et al. [42] in *Aloe vera* flowers harvested in south Sonora, México.

The fatty acids composition of *Aloe vera* fillet is presented in Table 2. Eighteen fatty acids were detected, with predominance of palmitic (C16:0, 32.1 ± 0.6%), stearic (C18:0, 16.4 ± 0.2%), linoleic (C18:2n6, 15.0 ± 0.2%), and oleic (C18:1n9, 12.9 ± 0.1%) acids. Thus, 67 ± 1% of the lipid fraction is constituted by saturated fatty acids (SFA) and 32.9 ± 0.4% corresponds to mono- and polyunsaturated (MUFA and PUFA) fatty acids. Essential PUFA such as C18:2n6 and linolenic acid (C18:3n3) play important biological functions and are involved in the modulation of inflammatory and chronic degenerative diseases [43]. Despite this, its concentration in the fillet is very low compared to other compounds identified with possible health-promoting effects. Odd-chain SFA, including pentadecanoic (C15:0) and heptadecanoic (C17:0) acids, were also found (Table 2). These two fatty acids have been gaining research interest because they are important as quantitative internal standards and biomarkers for assessing dietary food intake and the risk of coronary heart disease and type II diabetes mellitus [44]. A previous study also reported C18:2n6, C18:3n3, lauric acid (C12:0) and myristic acid (C14:0) in *Aloe vera* gel [45]. Regarding other *Aloe* species, the fatty acids C18:2n6, C16:0, lignoceric (C24:0), C18:0, tricosanoic (C23:0), behenic (C22:0), and C18:3n3, among others, were described in *Aloe ferox* gel [46]; and C18:3n3, C18:2n6, C16:0, C18:0, C18:1n9, and C14:0 were found in a whole leaf extract of *Aloe arborescence* [47].

### 3.2. Sugars and Glycosidic-linkage Composition of Fillet

Table 3 shows that the fillet sample contained 64 g/100 g dw of sugars, mainly glucose, uronic acids, and mannose. In this sample, 35 g/100 g dw of the sugars were found in the free form, determined as glucose and mannose (Table 1). The identification of the alditol acetate corresponding to mannose is probably due to the presence of fructose, as the methodology used converts fructose in glucitol (57%) and mannitol (43%) [48]. These results show that fillet sample contained 8 g/100 g dw of fructose and 27 g/100 g dw of glucose. A previous study reported fructose and glucose contents ranging from 0.56 to 9.62 g/100 g dw and 4.57 to 28.27 g/100 g dw, respectively, in fresh gel and powdered gel concentrates of *Aloe vera* [9], thus comprising the value quantified in this study.

Upon dialysis (12–14 kDa cut off), the weight of the obtained high molecular weight (HMW) material accounted for 39.9%. The sample obtained after dialysis (HMW) was composed by 77% of sugars, mainly mannose (65%), although glucose was also present (16%). To disclose the polysaccharides composition of the HMW sample, a methylation analysis was performed (Table 4).

The glycosidic-linkage composition of dialysed *Aloe vera* fillet shows a mannan composed by a backbone of (β1→4)-mannose residues [4,6], as observed by the high amount of the (1→4)-linked mannose residues (74.0%). Although (β1→4)-glucose residues may be present as insertions of the mannan backbone [49], these can have also indicate the presence of a glucan, possibly cellulose. *Aloe vera* mannan is also reported to be acetylated at the C-2 and C-3 positions and containing some side chains, mainly of galactose attached to C-6 [4,6]. The analysis performed using alkali conditions does not allow to maintain the acetyl groups. Nevertheless, the presence of (1→2,4)- and (1→3,4)-linked mannose residues are probably resultant from resistant acetylation positions of the mannan (Table 4). The branching percentage (3.2%), which can be estimated by the ratio between the (1→4,6)- mannose and the total amount of mannose, is in accordance with the presence of terminally-linked galactose and arabinose residues, identified in both pyranose (0.5%) and furanose (0.4%) forms [6]. The ratio calculated by the relative amount of total mannose divided by the amount of terminally linked mannose shows that this polysaccharide had a higher molecular weight than those previously reported [6].

### 3.3. Phenolic Composition of Fillet, Mucilage, Rind, and Flower

**Figure 1:**
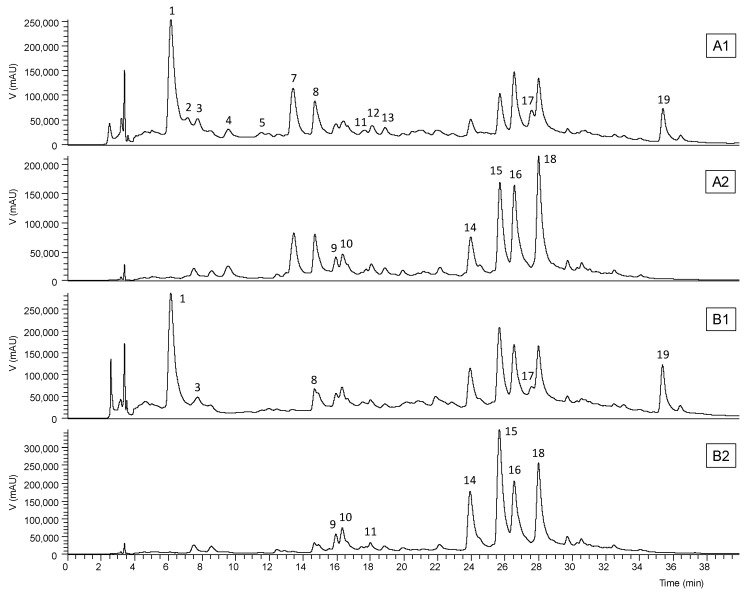
HPLC phenolic profiles of *Aloe vera* rind and mucilage extracts recorded at 280 nm (A1 and B1, respectively) and 370 nm (A2 and B2, respectively). See Table 5 for peak identification.

**Figure 2:**
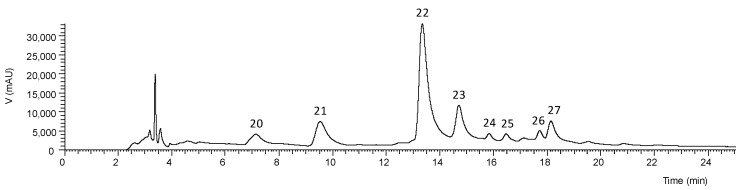
HPLC phenolic profile of *Aloe vera* flower extract recorded at 370 nm. See Table 5 for peak identification.

Data related to the phenolic compounds identification in the obtained *Aloe vera* extracts are presented in Table 5, namely the retention time, λ_max_ in the UV-vis region, pseudomolecular ion, ions of major fragments in MS^2^, and tentative identification (the obtained extraction yields and the phenolic contents that can be found in fresh and dried samples are shown in Table S1). The chromatographic profiles recorded at 280 and 370 nm are shown in Figure 1 and Figure 2. Up to 17 phenolic compounds were identified in the leaf extracts and eight in the flower extract, which were classified into four groups—phenolic acids, flavonoids, chromones, and anthrones. Most of these compounds have already been previously reported in *Aloe vera* [50,51,52,53], so that their identities were attributed by interpreting data acquired from HPLC-DAD-ESI/MS^n^ with those of literature.

The chromones aloesin or aloeresin B (peak 1) and 2’-*p*-methoxycoumaroylaloresin (peak 17) and the anthrones 10-hydroxyaloin B (peak 9), 10-hydroxyaloin A (peak 10), aloin B or isobarbaloin (peak 14), aloin A or barbaloin (peak 15), malonyl aloin B (peak 16), and malonyl aloin A (peak 18) were detected in the three studied parts of the *Aloe vera* leaf (Table 6). The mucilage contained the highest content (131 ± 3 mg/g extract) of phenolic compounds, mostly anthrones (62.1%) and chromones (34.6%), followed by two luteolin glucosides (3.3%). This result is in accordance with the literature, which states that the vascularized layer that covers the inner fillet is rich in anthraquinone glycosides and anthrone derivatives [3]. The rind was ranked second, with 105 ± 3 mg/g extract of phenolic compounds, of which 44.9% anthrones and 43.8% chromones; it also contained luteolin and apigenin glucosides and the phenolic acid *p*-coumaroylquinic acid. However, the chromone levels found in the rind did not differ statistically from those of the mucilage (Table 6). Although the phenolic profiles of the fillet and mucilage were similar, a significantly lower concentration (11.2 ± 0.2 mg/g extract) of these secondary metabolites was found in the fillet. In addition, this leaf part had an equal ratio of anthrones and chromones (Table 6).

Variations in the phenolic profiles of *Aloe* species have been reported. According to Fan et al. [50], aloesin is more abundant in *A. barbadensis* and *A. ferox* than in *A. chinensis* and *A. arborescens*. In these species, aloin A predominated over aloin B (according to our results), and lower concentrations were also found in *A. chinensis*. In general, higher contents and more complex phenolic compounds were reported in *A. barbadensis*. Kanama et al. [54] found minimal qualitative variations in the phenolic profiles of *A. ferox* exudate samples obtained from different regions of South Africa. Despite this, aloin B content varied from 18.4 to 149.7 mg/g, aloin A ranged from 21.3 to 133.4 mg/g, and aloesin from 111.8 to 561.8 mg/g of dried exudate. This result corroborates the data presented in Table 6, since aloesin predominated over both aloins, despite lower levels have been quantified in our samples.

6′-Malonylnataloin (peak 13), a malonylated derivative of the rare anthrone nataloin, was detected in the rind extract (Table 6). This anthrone *C*-glycoside is considered of great importance in systematic discrimination of different *Aloe* species and has been reported in *A. vera*, *A. arborescens*, *A. ellenbeckii*, *A. eru*, *A. grandidentata*, *A. brevifolia*, and *A. ferox* [52,55].

The quantification of aloins (as hydroxyanthracene derivatives) is recommended in routine quality control analyses of *Aloe* samples. These compounds are highly valorised in the pharmaceutical industry, allowed in dietary supplements, and used in small quantities as a bittering agent in alcoholic beverages. However, because of their laxative properties, levels of aloin A and B in *Aloe* leaf preparations intended for oral consumption were limited by the International Aloe Science Council to 10 ppm (10 mg/kg) or less [56]. These levels can be controlled and limited by adding purification steps in the manufacturing process.

The phenolic profile of *Aloe vera* flower (Figure 2) was different from that of leaf (Figure 1), being constituted mainly by the flavonoids apigenin-6,8-*C*-diglucoside, apigenin-2’’-*O*-pentoxide-*C*-hexoside, apigenin-6-*C*-glucoside, and traces of luteolin glucoside derivatives (accounting for 93.4% of the extract), and by the phenolic acid 5-*O*-caffeoylquinic acid (Table 5). As shown in Table 6, this extract had the lowest levels (4.78 ± 0.05 mg/g extract) of phenolic compounds. As far as we know, it is the first time that some of these compounds are described in *Aloe vera* flower. No anthraquinone glycosides were detected in this part of the plant as previously stated by Keyhanian and Stahl-Biskup [51].

### 3.4. Bioactive Properties of Fillet, Mucilage, Rind, and Flower

#### 3.4.1. Antioxidant Capacity

The results of the antioxidant capacity of the *Aloe vera* extracts are presented in Table 7. For the OxHLIA assay, data were given as IC_50_ values, corresponding to the extract concentration capable of protecting 50% of the erythrocyte population from oxidative haemolysis for 60 min; whereas, for the TBARS and β-CBI assays, data were expressed as EC_50_ values, meaning the extract concentration capable of providing 50% of antioxidant activity. In both cases, the lower the EC_50_ or IC_50_ values, the higher the antioxidant capacity. 

The mucilage extract (at 47 ± 2 µg/mL) was the most effective in inhibiting the formation of TBARS. This cell-based assay allowed for the measuring the extract capacity to inhibit the formation of malondialdehyde and other reactive substances, which are generated from the *ex vivo* decomposition of lipid peroxidation products resulting from the oxidation of the porcine brain cell membranes.

The rind extract provided the best protection against oxidative haemolysis (IC_50_ value, 56 ± 4 µg/mL) and β-carotene bleaching (EC_50_ value, 51 ± 4 µg/mL). In the OxHLIA assay, the erythrocytes were exposed to the haemolytic action of hydrophilic radicals that resulted from the thermal decomposition of the peroxyl radical initiator AAPH and, subsequently, to the action of lipophilic radicals generated through a lipid peroxidation phenomenon as a result of the initial attack [57]. In the β-CBI assay, β-carotene underwent discoloration in the absence of antioxidant extract, which results in a reduction in the absorbance of the test solution with increasing reaction time. The presence of antioxidants hindered the bleaching extension by neutralizing the linoleic hydroperoxyl radicals formed in the reaction emulsion [58]. These results are consistent with those of Lucini et al. [59], which concluded that the green rind is more antioxidant than the inner parenchyma.

Pearson’s analysis indicated a strong correlation between the antihaemolytic and β-carotene bleaching inhibition capabilities and the flavonoids content (*r* = −0.778, *p* = 0.003, and *r* = −0.865, *p* < 0.001, respectively). In fact, the higher IC_50_ and EC_50_ values obtained with these two *in vitro* assays, respectively, were achieved with fillet extract (Table 7), in which no flavonoids were detected (Table 6). On the other hand, TBARS inhibition was strongly correlated with malic acid contents (*r* = −0.946, *p* <0.001) and moderately with the levels of anthrones (*r* = −0.667, *p* = 0.018) and chromones (*r* = −0.676, *p* = 0.016). The results of a previous work [60] suggested that the antioxidant capacity of *Aloe vera* gel extract might be ascribed to a synergistic action of bioactive compounds. The same study also evidenced that the gel extract is able to protect the erythrocyte membrane from AAPH-induced oxidative injury and partially restore its normal protein profiles. This observation may support the fact that the best results of the antioxidant activity of the present study were achieved with the OxHLIA assay because the extracts had IC_50_ values closer to those of trolox.

#### 3.4.2. Antimicrobial Activity

Since *Aloe vera* has been used in the development of topical cosmeceutical formulations, microorganisms of the skin flora were also tested. *Staphylococcus aureus*, *S. epidermidis*, and *S. lugdunensis* are commensal bacteria of the skin that can become opportunistic pathogens and cause a number of skin infections and also life-threatening diseases [61,62]. For these three bacteria, MIC and MBC values ≤ 0.6 and 0.8 mg/mL, respectively, were reached with the tested extracts (Table 7). The mucilage extract was the most effective against *S. lugdunensis* (with MIC and MBC values of 0.2 and 0.4 mg/mL, respectively), a Gram-positive bacterium that has been associated with a wide variety of infections, including skin and soft-tissue infections (furuncles, cellulitis, and abscesses), but also cardiovascular, central nervous infections, and urinary tract infections [63]. The mucilage extract was also effective in inhibiting and killing the *Micrococcus flavus* and also *Listeria monocytogenes* (Table 7), a food-borne pathogen capable of infecting both humans and animals. Occasionally, *L. monocytogenes* can cause localized skin infections, especially in people dealing with infected animals. The activity against this pathogenic bacterium was found strongly correlated with anthrones (*r* = −0.845 for MIC and *r* = −0.816 for MBC, *p* = 0.001) and chromones (*r* = −0.795 for MIC and *r* = −0.861 for MBC, *p* ≤ 0.002) and moderately/strongly with flavonoids (*r* = −0.611, *p* = 0.035, for MIC and *r* = −0.781, *p* ≤ 0.003, for MBC).

The rind extract had the best results against *Salmonella* Typhimurium (*S. enterica* serovar Typhimurium) (MIC of 0.4 mg/mL and MBC of 0.8 mg/mL) and rind and flower extracts against *Escherichia coli* (MIC of 0.025 mg/mL and MBC of 0.05 mg/mL). In the case of *Pseudomonas aeruginosa*, the flower extract was as effective as the antibiotic streptomycin in inhibiting (0.025 mg/mL) and killing (0.05 mg/mL) this multidrug resistant opportunistic pathogen. Interestingly, this carbapenem-resistant Gram-negative pathogen appears in the 2017 WHO list of threatening bacteria for which new antibiotics are urgently needed [64].

As presented in Table 7, three of the *Aloe vera* extracts (except for mucilage) had an antifungal activity (MIC and MFC values ≤ 0.1 and 0.4 mg/mL, respectively) against *Aspergillus flavus*, *A. niger*, and *Penicillium funiculosum* superior to that of the positive control ketoconazole (MIC and MFC values ≤ 0.25 and 0.5 mg/mL, respectively). All extracts were more effective than ketoconazole in inhibiting (MIC, 0.05 mg/mL) or killing (MFC, 0.1 mg/mL) the opportunistic yeast *Candida albicans*, which is the most prevalent fungal pathogen in humans, causing candidiasis. This fungal infection affects predominantly superficial skin and mucosa (oral and vaginal), but it can also lead to life-threatening systemic infections [65].

Filamentous fungi causing superficial infections in keratinized tissues, namely *Trichophyton mentagrophytes*, *T. tonsurans*, *Microsporum gypseum*, and *M. canis*, were also tested. As found for the other microorganisms, extract concentrations ≤ 0.2 and 0.4 mg/mL were sufficient to inhibit growth or kill, respectively, these dermatophytes (Table 7). The rind extract was the most effective against *T. tonsurans*, while the flower followed by the fillet extracts were the most effective against *M. gypseum* and *M. canis*.

#### 3.4.3. Tyrosinase Inhibition Capacity

The extracts capacity to inhibit tyrosinase activity can be translated into their potential as skin whitening and anti-hyperpigmentation agents, since melanin production is impaired when this enzyme is inhibited, resulting in a less pigmented skin [66]. In this study, the flower extract was the most active (with an IC_50_ of 4.85 ± 0.07 mg/mL), but still not as effective as kojic acid (IC_50_ of 0.078 ± 0.001 mg/mL), the depigmenting agent used as a positive control (Table 7). The high percentage of flavonoids present in this extract (Table 6) may justify the observed inhibitory effect. Previous studies reported that plant extracts rich in flavonoids have a strong suppressive impact on tyrosinase, which enables its use in skin lightening cosmeceuticals [66]. For rind and mucilage extracts, it was not possible to calculate IC_50_ values, so the results were given as percentage of inhibition. The fillet did not provide any value in terms of tyrosinase inhibition. Despite this, chromones isolated from *Aloe vera*, including aloesin that was found mainly in rind and mucilage, have been reported to have tyrosinase inhibitory activity [14].

#### 3.4.4. Anti-Inflammatory and Cytotoxic Activities

Although it is claimed that *Aloe vera* gel has important therapeutic properties, the performed bioassays indicated that none of the four extracts has anti-inflammatory activity or cytotoxicity against metastatic melanoma (MM127), breast adenocarcinoma (MCF-7), non-small cell lung carcinoma (NCI-H460), cervical carcinoma (HeLa), and hepatocellular carcinoma (HepG2) at the tested concentrations (EC_50_ and GI_50_ values >400 μg/mL). Hepatotoxicity was also not observed against the non-tumour PLP2 cell line, whereas a GI_50_ value of 8.6 ± 0.1 μg/mL was obtained for ellipticine, the anticancer agent used as positive control. These results are somewhat supported by the study of du Plessis and Hamman [67], in which the cytotoxic activity of *Aloe vera*, *Aloe marlothii*, *Aloe speciosa*, and *Aloe ferox* gels was investigated against HepG2, HeLa, and human neuroblastoma (SH-SY5Y) cells. A decrease in cell viability was reported only at concentrations >10 mg/mL, and the half-maximal cytotoxic concentration (CC_50_) values were above 1000 mg/mL, except for *Aloe vera* gel in HepG2 cells (CC_50_ value = 269.3 mg/mL). Hussain et al. [68] reported that *Aloe vera* gel (extracted from dried leaves and used directly as a drug solution) displays cytotoxic effects against the MCF-7 and HeLa cell lines by inducing apoptosis and modulating the expression of effector molecules. In addition, no significant cytotoxicity toward normal lymphocytes was recorded for 24 h.

Most of the cytotoxicity studies available in the literature report the use of isolated compounds rather than crude extracts, which consist of a mixture of phytochemicals and other constituents with or without bioactive properties. El-Shemy et al. [69] tested aloesin, aloe-emodin, and barbaloin extracted from *Aloe vera* leaves against Ehrlich ascite carcinoma and found a significant inhibition as follows: barbaloin >aloe-emodin >aloesin. The authors also described that aloe-emodin was active against the human colon cancer cell lines DLD-1 and HT2. The antimetastatic potential of aloe-emodin on highly metastatic B16-F10 melanoma murine cells has also been described [70].

## 4. Conclusions

This study showed that 58% of the total *Aloe vera* leaf weight corresponds to inner fillet, but the green rind also accounted for a considerable percentage. The fillet consisted mainly of moisture, followed by dietary fibre and available carbohydrates (mainly glucose and fructose). Malic acid, which is an excellent freshness indicator, and α-tocopherol, a powerful fat-soluble chain-breaking antioxidant, were detected in high amounts in this jelly-like parenchyma. Based on the glycosidic-linkage composition, it was concluded that the fillet sample is composed mainly by mannans, possibly acemannan.

The three leaf samples revealed similar phenolic profiles, with predominance of chromones (aloesin and 2’-*p*-methoxycoumaroylaloresin) and anthrones (aloin A and B, malonyl aloin A and B, and 10-hydroxyaloin A and B). The highest contents of phenolic compounds were found in the mucilage and rind extracts, which also revealed interesting antioxidant properties. On the other hand, the flower extract was rich in apigenin glycoside derivatives, effective against the multidrug-resistant *P. aeruginosa*, and capable of inhibiting the activity of the enzyme tyrosinase. The fillet, rind, and flower extracts also showed a powerful antifungal activity against *A. flavus*, *A. niger*, *P. funiculosum*, and *C. albicans*, higher than that of ketoconazole. Therefore, all the studied *Aloe vera* samples present high potential to be exploited by food or cosmeceutical industries.

## Figures and Tables

**Table 1:** Nutritional value and organic acids and tocopherols composition of *Aloe vera* fillet.

Nutritional Component	Fresh Fillet	Dry Powder
Moisture (g/100 g) ^1^	98 ± 1	-
Protein (g/100 g)	0.044 ± 0.001	2.60 ± 0.05
Ash (g/100 g)	0.150 ± 0.003	9.0 ± 0.2
Fat (g/100 g)	0.0168 ± 0.0006	1.00 ± 0.04
Available carbohydrates (g/100 g)	0.630 ± 0.006	37.4 ± 0.3
Dietary fibre (g/100 g)	0.84 ± 0.02	50.1 ± 0.3
Crude fibre (g/100 g)	0.120 ± 0.003	7.1 ± 0.2
Energy (kcal/100 g)	4.54 ± 0.05	269 ± 3
Oxalic acid (mg/100 g)	2.39 ± 0.04	142 ± 2
Quinic acid (mg/100 g)	11.63 ± 0.07	689 ± 4
Malic acid (mg/100 g)	97 ± 1	5750 ± 66
Total organic acids (mg/100 g)	111 ± 1	6581 ± 73
α-Tocopherol (µg/100 g)	81 ± 2	4813 ± 104
β-Tocopherol (µg/100 g)	3.59 ± 0.06	213 ± 3
γ-Tocopherol (µg/100 g)	6.7 ± 0.1	396 ± 8
δ-Tocopherol (µg/100 g)	1.78 ± 0.02	106 ± 1
Total tocopherols (µg/100 g)	93 ± 2	5527 ± 98

^1^ The results are presented as mean ± standard deviation.

**Table 2:** Fatty acids composition of *Aloe vera* fillet.

Fatty Acid	Relative Percentage (%) ^1^
Caproic acid (C6:0)	0.51 ± 0.01
Caprylic acid (C8:0)	0.21 ± 0.01
Capric acid (C10:0)	0.70 ± 0.01
Lauric acid (C12:0)	6.83 ± 0.09
Myristic acid (C14:0)	3.57 ± 0.06
Pentadecanoic acid (C15:0)	0.41 ± 0.02
Palmitic acid (C16:0)	32.1 ± 0.6
Heptadecanoic acid (C17:0)	0.92 ± 0.02
Stearic acid (C18:0)	16.4 ± 0.2
Oleic acid (C18:1n9c)	12.9 ± 0.1
Linoleic acid (C18:2n6c)	15.0 ± 0.2
α-Linolenic acid (C18:3n3)	4.00 ± 0.07
Arachidic acid (C20:0)	0.689 ± 0.008
Heneicosanoic acid (C21:0)	0.212 ± 0.002
Behenic acid (C22:0)	1.14 ± 0.01
Erucic acid (C22:1)	0.95 ± 0.01
Tricosanoic acid (C23:0)	0.86 ± 0.04
Lignoceric acid (C24:0)	2.54 ± 0.01
Saturated fatty acids (SFA)	67 ± 1
Monounsaturated fatty acids (MUFA)	13.8 ± 0.04
Polyunsaturated fatty acids (PUFA)	19.0 ± 0.2

^1^ The results are presented as mean ± standard deviation.

**Table 3:** Carbohydrates of the initial and dialysed *Aloe vera* fillet identified as alditol acetates.

Fillet Sample	Carbohydrate (mol%)						Total Carbohydrate	
	Ara	Xyl	Man	Gal	Glc	UA	(g/100 g)	RSD (%)
**Initial sample**								
**Total sugars**	1.0	2.0	21.4	3.5	50.1	22.0	64.0	3
**Free sugars**			9.7		90.3		34.9	12
**HMW**	1.4	1.5	65.2	3.2	15.9	12.7	76.5	6

HMW: high molecular weight (sample dialysed with a membrane cut-off of 12–14 kDa). Ara: arabinose; Xyl: xylose; Man: mannose; Gal: galactose; Glc: glucose; UA: uronic acids; RSD: relative standard deviation.

**Table 4:** Glycosidic-linkage composition of the dialysed *Aloe vera* fillet.

Glycosyl Linkage	HMW (> 14 kDa)	
	% mol	RSD (%)
*t*-Ara*f*	0.4	7
*t*-Ara*p*	0.5	24
5-Ara*f*	0.7	2
**Total**	**1.7**	**10**
*t*-Xyl*p*	0.2	9
4-Xylp	1.7	21
***Total***	**1.9**	**20**
*t*-Man	1.0	16
4-Man	74.0	0
2,4-Man	1.6	36
3,4-Man	1.1	35
4,6-Man	2.6	12
**Total**	**80.2**	**2**
*t*-Gal	0.5	28
**Total**	**0.5**	**28**
*t*-Glc	0.6	3
4-Glc	14.0	7
4,6-Glc	1.1	17
**Total**	**15.6**	**8**
**Total Man*p*/T-Man*p***	77.4	
**% Branching**	3.2	

HMW: high molecular weight (sample dialysed with a membrane cut-off of 12–14 kDa). RSD: relative standard deviation.

**Table 5:** Phenolic compounds identified in *Aloe vera* leaf and flower extracts. Retention time (Rt), wavelengths of maximum absorption in the UV-vis region (λmax), pseudomolecular and MS^2^ fragment ions, and relative abundance in brackets.

Peak	Rt (min)	λ_max_ (nm)	[M−H]^−^ (*m/z*)	MS^2^ (*m/z*)	Tentative Identification ^1^	Reference
**Part A:***Aloe vera* leaf (fillet, mucilage and rind)						
1	6.14	227, 245, 252, 298	393	375(3), 303(12), 273(100), 245(5), 203(3)	Aloesin (aloeresin B) isomer 1	[50]
2	7.13	213, 243, 252, 299	393	375(3), 303(14), 273(100), 245(4), 203(3)	Aloesin (aloeresin B) isomer 2	[50]
3	7.73	213, 244, 252, 299	455	473(5), 411(5), 391(7), 365(100), 341(3), 333(11), 275(3), 243(3)	Unknown	-
4	9.56	254, 271, 345	609	447(100), 357(5), 327(15)	Luteolin-6,8-*C*-diglucoside	[51]
5	11.53	306	337	191(100), 173(5), 163(10), 119(3)	*cis* 5-*p*-Coumaroylquinic acid	[51]
6	12.63	306	337	191(100), 173(5), 163(10), 119(3)	*trans* 5-*p*-Coumaroylquinic acid	[51]
7	13.41	270, 340	593	473(43), 431(100), 311(79)	Apigenin-6,8-*C*-diglucoside	[52]
8	14.71	254, 271, 346	447	357(52), 327(100)	Luteolin-6-*C*-glucoside	[52]
9	15.98	222, 272, 303, 355	433	343(3), 313(100), 271(5), 255(3)	10-Hydroxyaloin B	[50]
10	16.36	222, 273, 305, 355	433	343(3), 313(100), 271(5), 255(3)	10-Hydroxyaloin A	[50]
11	17.60	227, 272, 301, 350	433	343(5), 313(37), 271(100), 255(3)	5-Hydroxyaloin A	[52]
12	18.11	272, 340	431	413(8), 341(27), 311(100)	Apigenin-6-*C*-glucoside	[52]
13	18.88	219, 269, 301, 357	505	448(3), 343(100), 172(5)	6′-Malonylnataloin	[52]
14	23.97	225, 269, 298, 355	417	297(100), 255(3)	Aloin B (isobarbaloin)	[50]
15	25.71	225, 269, 298, 355	417	297(100), 255(3)	Aloin A (barbaloin)	[50]
16	26.56	225, 269, 298, 355	503	459(100), 417(5), 297(40)	Malonyl aloin B	-
17	27.62	229, 252, 300	553	407(100), 375(5), 347(11), 275(52), 259(5), 233(7), 191(4)	2’-*p*-Methoxycoumaroylaloresin	[52]
18	28.01	225, 269, 298, 355	503	459(100), 417(5), 297(48)	Malonyl aloin A	-
19	36.41	252, 284	585	567(5), 521(18), 495(100), 463(3), 373(3), 333(3)	Unknown	-
**Part B:***Aloe vera* flower						
20	7.13	298, 324	353	191(100), 179(10), 173(5), 135(3)	5-*O*-Caffeoylquinic acid	[52]
21	9.53	254, 271, 345	609	447(100), 357(5), 327(15)	Luteolin-6,8-*C*-diglucoside	[52]
22	13.34	270, 340	593	473(43), 431(100), 311(79)	Apigenin-6,8-*C*-diglucoside isomer 1	[52]
23	14.71	254, 271, 346	447	357(52), 327(100)	Luteolin-6-*C*-glucoside	[52]
24	15.80	270, 340	593	473(43), 431(100), 311(79)	Apigenin-6,8-*C*-diglucoside isomer 2	[52]
25	16.50	271, 340	563	443(7), 413(100), 323(5), 311(3), 293(27)	Apigenin-2’’-*O*-pentoxide-*C*-hexoside	[53]
26	17.70	-	593	473(8), 443(100), 371(3), 353(3), 341(3), 323(28), 311(9), 285(3)	Methyl-luteolin-2’’-*O*-pentoxide-*C*-hexoside	[53]
27	18.14	272, 340	431	413(8), 341(27), 311(100)	Apigenin-6-*C*-glucoside	[52]

^1^ The compounds identity was attributed by interpreting data acquired from HPLC-DAD-ESI/MS^n^ with those of the literature.

**Table 6:** Content of phenolic compounds in *Aloe vera* leaf (fillet, mucilage, and rind) and flower extracts. See Table 5 for peak identification.

Peak	Content (mg/g Extract) ^1^				Statistics	
	Fillet	Mucilage	Rind	Flower	H ^2^	*p*-Value ^3^
1	5.4 ± 0.4 ^c^	39 ± 3 ^a^	34.4 ± 0.7 ^b^	-	0.176	<0.001
2	-	-	4.8 ± 0.2	-	-	-
4	-	3.5 ± 0.2	tr	-	-	-
5	0.180 ± 0.008	-	0.31 ± 0.01	-	0.560	<0.001*
6	0.145 ± 0.002	-	-	-	-	-
7	-	-	6.67 ± 0.04	-	-	-
8	tr	0.79 ± 0.01 ^b^	3.3 ± 0.1 ^a^	-	0.095	<0.001
9	tr	3.2 ± 0.2 ^a^	2.83 ± 0.06 ^b^	-	0.120	<0.001
10	tr	5.1 ± 0.4 ^a^	3.8 ± 0.2 ^b^	-	0.149	<0.001
11	-	3.35 ± 0.06	2.4 ± 0.2	-	0.286	<0.001*
12	-	-	1.64 ± 0.05	-	-	-
13	-	-	3.23 ± 0.04	-	-	-
14	0.24 ± 0.03 ^c^	13.3 ± 0.1 ^a^	4.3 ± 0.3 ^b^	-	0.240	<0.001
15	1.24 ± 0.06 ^c^	22.2 ± 0.5 ^a^	9.9 ± 0.4 ^b^	-	0.292	<0.001
16	1.43 ± 0.08 ^c^	16.6 ± 0.4 ^a^	7.8 ± 0.4 ^b^	-	0.350	<0.001
17	0.13 ± 0.04 ^c^	6.08 ± 0.08 ^b^	6.9 ± 0.4 ^a^	-	0.139	<0.001
18	2.4 ± 0.1 ^c^	17.6 ± 0.2 ^a^	12.8 ± 0.8 ^b^	-	0.157	<0.001
20	-	-	-	0.30 ± 0.01	-	-
21	-	-	-	tr	-	-
22	-	-	-	2.42 ± 0.04	-	-
23	-	-	-	tr	-	-
24	-	-	-	0.94 ± 0.01	-	-
25	-	-	-	0.96 ± 0.01	-	-
26	-	-	-	tr	-	-
27	-	-	-	0.152 ± 0.009	-	-
**Σ Phenolic acids**	0.33 ± 0.01 ^a^	-	0.31 ± 0.01 ^a,b^	0.30 ± 0.01 ^b^	0.735	0.022
**Σ Flavonoids**	-	4.3 ± 0.2 ^b^	11.6 ± 0.1 ^a^	4.48 ± 0.05 ^b^	0.298	<0.001
**Σ Anthrones**	5.3 ± 0.3 ^c^	81 ± 1 ^a^	47 ± 2 ^b^	-	0.291	<0.001
**Σ Chromones**	5.5 ± 0.5 ^b^	45 ± 3 ^a^	46 ± 1 ^a^	-	0.259	<0.001
**Σ Phenolic compounds**	11.2 ± 0.2 ^c^	131 ± 3 ^a^	105 ± 3 ^b^	4.78 ± 0.05 ^d^	0.134	<0.001

tr: traces. ^1^ The results are presented as mean ± standard deviation. ^2^ Homoscedasticity (H) was tested by the Levene’s test: *p* > 0.05 indicates homoscedasticity and *p* < 0.05 indicates heteroscedasticity. ^3^ Statistically significant differences (*p* < 0.05) among two samples* were assessed by a Student’s *t*-test and among more than two samples were assessed by a one-way ANOVA (and indicated by different letters), using Tukey’s honestly significant difference (HSD) or Tamhane’s T2 multiple comparison tests, when homoscedasticity was verified or not, respectively.

**Table 7:** Antioxidant, anti-tyrosinase, and antimicrobial capacities of *Aloe vera* leaf (fillet, mucilage, and rind) and flower extracts and positive controls.

	Fillet		Mucilage		Rind		Flower		Positive Control	
**Antioxidant Activity** ^1^									Trolox	
OxHLIA (IC_50_, µg/mL)	378 ± 18 ^a^		105 ± 8 ^b^		56 ± 4 ^c^		80 ± 4 ^b,c^		20.4 ± 0.4 ^d^	
TBARS (EC_50_, µg/mL)	87 ± 4 ^b^		47 ± 2 ^c^		97 ± 3 ^b^		347 ± 14 ^a^		5.4 ± 0.3 ^d^	
β-CBI (EC_50_, µg/mL)	78 ± 6 ^a^		63 ± 4 ^b^		51 ± 4 ^c^		59 ± 4 ^b,c^		0.20 ± 0.01 ^d^	
**Anti-Tyrosinase Activity**									Kojic Acid	
IC_50_ (mg/mL) or I(%) ^2^	na		30.38 ± 0.01%		27.2 ± 0.7%		4.85 ± 0.07		0.078 ± 0.001	
**Antibacterial Activity**									Streptomycin	
	MIC	MBC	MIC	MBC	MIC	MBC	MIC	MBC	MIC	MBC
*Staphylococcus aureus*	0.60	0.80	0.60	0.80	0.60	0.80	0.60	0.80	0.006	0.012
*Staphylococcus epidermidis*	0.60	0.80	0.40	0.80	0.40	0.80	0.60	0.80	0.003	0.006
*Staphylococcus lugdunensis*	0.40	0.80	0.20	0.40	0.40	0.80	0.40	0.80	0.025	0.050
*Micrococcus flavus*	0.80	1.60	0.60	0.80	0.80	1.20	0.40	0.80	0.20	0.30
*Listeria monocytogenes*	1.20	1.60	0.40	0.80	0.60	0.80	0.80	1.20	0.20	0.30
*Escherichia coli*	0.050	0.10	0.10	0.20	0.025	0.050	0.025	0.050	0.006	0.012
*Pseudomonas aeruginosa*	0.10	0.20	0.050	0.10	0.10	0.20	0.025	0.050	0.025	0.050
*Salmonella* Typhimurium	0.80	1.20	0.80	1.20	0.40	0.80	0.80	1.20	0.25	0.50
**Antifungal Activity**									Ketoconazole	
	MIC	MFC	MIC	MFC	MIC	MFC	MIC	MFC	MIC	MFC
*Aspergillus flavus*	0.10	0.20	0.80	>1.60	0.10	0.20	0.10	0.20	0.25	0.50
*Aspergillus niger*	0.10	0.20	>1.60	>1.60	0.20	0.40	0.10	0.20	0.20	0.50
*Penicillium funiculosum*	0.050	0.10	>1.60	>1.60	0.050	0.10	0.10	0.20	0.20	0.50
*Candida albicans*	0.050	0.10	0.050	0.10	0.050	0.10	0.050	0.10	0.40	0.80
*Trichophyton mentagrophytes*	0.10	0.20	0.10	0.20	0.20	0.40	0.10	0.20	0.012	0.025
*Trichophyton tonsurans*	0.025	0.050	0.025	0.050	0.012	0.025	0.050	0.10	0.0015	0.003
*Microsporum gypseum*	0.025	0.050	0.20	0.40	0.050	0.10	0.012	0.025	0.006	0.012
*Microsporum canis*	0.025	0.050	0.050	0.10	0.050	0.10	0.025	0.050	0.003	0.006

na: no activity; MIC: minimum inhibitory concentration (mg/mL); MBC: minimum bactericidal concentration (mg/mL); MFC: minimum fungicidal concentration (mg/mL). ^1^ Statistics for antioxidant activity: (^a^) homoscedasticity was tested by the Levene’s test: *p* = 0.130 for OxHLIA (homoscedastic); *p* = 0.010 for TBARS (heteroscedastic); and *p* = 0.305 for β-CBI (homoscedastic); and (^b^) Statistically significant differences (*p* < 0.05) were assessed by a one-way ANOVA (and indicated by different letters) using Tukey’s honestly significant difference (HSD) or Tamhane’s T2 multiple comparison tests, when homoscedasticity was verified or not, respectively: *p* < 0.001 in all cases. ^2^ Inhibition percentage of tyrosinase activity at 8 mg/mL.

## Supplementary Materials

The following are available online at https://www.mdpi.com/2076-3921/8/10/444/s1, Table S1. Moisture, organic acids and phenolic compounds composition of *Aloe vera* leaf (fillet, mucilage, and rind) and flower.
