# Supplementary material for: Compositional Features and Bioactive Properties of Aloe vera Leaf (Fillet, Mucilage, and Rind) and Flower
Source: Antioxidants (Basel). 2019 Oct 1;8(10):444. doi: 10.3390/antiox8100444 (PMC6826699; doi:10.3390/antiox8100444)
Supplement: Supplementary file 1 [file antioxidants-08-00444-s001.pdf]

Supplementary Material

Table S1: Moisture, organic acids and phenolic compounds composition of *Aloe vera* leaf (fillet, mucilage, and rind) and flower.

	Fillet	Mucilage	Rind	Flower	H[1]	*p*-value[2]
Moisture (g/100 g)	98 ± 1 [a]	98 ± 1 [a]	87 ± 1 [b]	84 ± 1 [b]	0.320	<0.001
Organic acids (mg/100 g fresh weight)						
Oxalic acid	2.39 ± 0.04 [a]	0.23 ± 0.01 [c]	0.76 ± 0.03 [b]	2.3 ± 0.1 [a]	0.172	<0.001
Quinic acid	11.63 ± 0.07	10.3 ± 0.2	-	-	0.354	0.001
Malic acid	97 ± 1 [b]	101 ± 2 [a]	58 ± 1 [c]	-	0.852	<0.001
Ascorbic acid	-	-	0.874 ± 0.007	-	-	-
Citric acid	-	-	11.2 ± 0.2	-	-	-
Fumaric acid	-	-	0.051 ± 0.004	0.540 ± 0.005	0.662	<0.001
Total organic acids	111 ± 1 [a]	111 ± 2 [a]	71 ± 2 [b]	2.9 ± 0.1 [c]	0.394	<0.001
Organic acids (mg/100 g dry weight)						
Oxalic acid	142 ± 2 [a]	13.7 ± 0.6 [c]	45 ± 2 [b]	139 ± 6 [a]	0.172	<0.001
Quinic acid	689 ± 4	610 ± 9	-	-	0.354	0.001
Malic acid	5750 ± 66 [b]	5979 ± 100 [a]	3462 ± 82 [c]	-	0.852	<0.001
Ascorbic acid	-	-	51.8 ± 0.4	-	-	-
Citric acid	-	-	659 ± 12	-	-	-
Fumaric acid	-	-	3.1 ± 0.2	32.1 ± 0.3	0.662	<0.001
Total organic acids	6581 ± 73 [a]	6603 ± 92 [a]	4221 ± 92 [b]	171 ± 8 [c]	0.394	<0.001
Extraction yield (%)	36.66	59.98	34.25	44.32	-	-
Phenolic compounds (μg/g fresh weight)						
Phenolic acids	1.97 ± 0.06 [d]	53 ± 3 [a]	13.8 ± 0.6 [c]	20.9 ± 0.5 [b]	0.102	<0.001
Flavonoids	-	-	523 ± 5	309 ± 4	0.736	<0.001
Anthrones	32 ± 2 [c]	1010 ± 12 [b]	2120 ± 75 [a]	-	0.109	<0.001
Chromones	34 ± 3 [c]	563 ± 34 [b]	1856 ± 48 [a]	-	0.238	<0.001
Phenolic compounds	68 ± 1 [d]	1626 ± 43 [b]	4513 ± 127 [a]	330 ± 3 [c]	0.091	<0.001
Phenolic compounds (mg/g dry weight)						
Phenolic acids	0.119 ± 0.004 [b]	2.6 ± 0.1 [a]	0.105 ± 0.005 [b]	0.134 ± 0.003 [b]	0.059	<0.001
Flavonoids	-	-	3.99 ± 0.04	1.98 ± 0.02	0.578	<0.001
Anthrones	2.0 ± 0.1 [c]	48.8 ± 0.6 [a]	16.2 ± 0.6 [b]	-	0.332	<0.001
Chromones	2.0 ± 0.2 [c]	27 ± 2 [a]	14.1 ± 0.4 [b]	-	0.146	<0.001
Phenolic compounds	4.11 ± 0.07 [c]	79 ± 2 [a]	34 ± 1 [b]	2.12 ± 0.02 [c]	0.105	<0.001

[1] Homoscedasticity (H) was tested by the Levene's test: $p > 0.05$ indicates homoscedasticity and $p < 0.05$ indicates heteroscedasticity. [2] Statistically significant differences ($p < 0.05$) among two samples were assessed by a Student's T-Test and among more than two samples were assessed by a one-way ANOVA, using Tukey's honestly significant difference (HSD) or Tamhane's T2 multiple comparison tests, when homoscedasticity was verified or not, respectively.
